# miR-195 Inhibits EMT by Targeting FGF2 in Prostate Cancer Cells

**DOI:** 10.1371/journal.pone.0144073

**Published:** 2015-12-09

**Authors:** Chunhui Liu, Han Guan, Yiduo Wang, Ming Chen, Bin Xu, Lei Zhang, Kai Lu, Tao Tao, Xiaowen Zhang, Yeqing Huang

**Affiliations:** 1 Department of Urology, Affiliated Zhongda Hospital of Southeast University, 87 Dingjia Bridge Hunan Road, Nanjing, 210009, China; 2 Surgical Research Center, Institute of Urology, Southeast University Medical School, 87 Dingjia Bridge Hunan Road, Nanjing, 210009, China; University of Texas MD Anderson Cancer Center, UNITED STATES

## Abstract

Prostate cancer (PCa) is one of the leading causes of deaths in America. The major cause of mortality can be attributed to metastasis. Cancer metastasis involves sequential and interrelated events. miRNAs and epithelial-mesenchymal transition (EMT) are implicated in this process. miR-195 is downregulated in many human cancers. However, the roles of miR-195 in PCa metastasis and EMT remain unclear. In this study, data from Memorial Sloan Kettering Cancer Center (MSKCC) prostate cancer database were re-analysed to detect miR-195 expression and its roles in PCa. miR-195 was then overexpressed in castration-resistant PCa cell lines, DU-145 and PC-3. The role of miR-195 in migration and invasion in vitro was also investigated, and common markers in EMT were evaluated through Western blot analysis. A luciferase reporter assay was conducted to confirm the target gene of miR-195; were validated in PCa cells. In MSKCC data re-analyses, miR-195 was poorly expressed in metastatic PCa; miR-195 could be used to diagnose metastatic PCa by measuring the corresponding expression. Area under the receiver operating characteristic curve (AUC-ROC) was 0.705 (P = 0.017). Low miR-195 expression was characterised with a shorter relapse-free survival (RFS) time. miR-195 overexpression suppressed cell migration, invasion and EMT. Fibroblast growth factor 2 (FGF2) was confirmed as a direct target of miR-195. FGF2 knockdown also suppressed migration, invasion and EMT; by contrast, increased FGF2 partially reversed the suppressive effect of miR-195. And data from ONCOMINE prostate cancer database showed that PCa patients with high FGF2 expression showed shorter RFS time (P = 0.046). Overall, this study demonstrated that miR-195 suppressed PCa cell metastasis by downregulating FGF2. miR-195 restoration may be considered as a new therapeutic method to treat metastatic PCa.

## Introduction

Prostate cancer (PCa), one of the leading causes of deaths in America, was responsible for 29,480 American deaths in 2014 [1]. Local primary tumor is rarely fatal. The major cause of mortality can be attributed to metastasis [2]. Approximately 90% of deaths from solid tumors are caused by metastasis [3]. PCa metastasis is found in <5% of patients in the first diagnosis. In castration-resistant PCa (CRPC) group, 50%–70% of the patients likely develop bone metastasis [4]. Therefore, the mechanism of PCa metastasis, especially CRPC, should be investigated to treat PCa.

Cancer metastasis involves sequential and interrelated events [5]. Epithelial-mesenchymal transition (EMT), known to turn epithelial cells into mesenchymal cells, also performs crucial functions in cancer metastasis [6]. Epithelial cells obtain mesenchymal cell characteristics, including acquisition of cell migration and invasion abilities, through EMT [7]. The mechanisms of EMT are complex. Many factors, including miRNAs [8], are associated with EMT. miRNAs are small, non-coding RNAs of 20–22 nt that posttranscriptionally modulate gene expression by binding to the 3′-untranslated region (3′-UTR) of mRNAs). Numbers of miRNAs are found be aberrant expresaion in cancer and implicate apoptosis, proliferation, differentiation and metastasis [9]. It is known that many miRNAs promote or inhibit metastatic tumor progression by regulating EMT [10]. miR-29b and miR-30a repressed the expression of master transcription factor Snail 1 in the programe of EMT [11, 12]. Therefore, increased miR-29b expression can inhibit EMT and decrease cell invasion [11]. Furthermore, miR-200 family members and miR-205 repress the translation of zinc-finger E-box-bindings (ZEBs) 1 and 2; ZEB 1 and ZEB2 expressions are activated early in EMT [13].

miR-195 belongs to the miR-15/16/195 family; this miRNA is localised inn chromosome 17p13.1. Aberrant miR-195 expression has been observed in many types of malignant cancers, such as breast, gastric, colon, adrenocortical, bladder and ovarian cancers, hepatocellular carcinoma and non-small cell lung cancer (NSCLC) [14–21]. Moreover, miR-195 can also inhibit invasion and migration in NSCLC, colorectal cancer and osteosarcoma [17, 18, 22]. miR-195 was also found be low expression in PCa tissues [23]. However, this study only analyzed miR-195 expression in prostate cancer, the effects of miR-195 on PCa pathobiology, especially in metastasis, are currently undetermined. So we investigate the role of miR-195 in EMT and metastasis of PCa in t the current study.

In this study, data from Memorial Sloan Kettering Cancer Center (MSKCC) prostate cancer database were re-analysed; results revealed that miR-195 was poorly expressed in metastatic PCa. Patients with lower miR-195 expression exhibited shorter relapse-free survival (RFS) time. miR-195 could also be used to diagnose metastatic PCa by measuring their corresponding expression; area under the receiver-operating characteristic curve (AUC-ROC) was 0.705 (P = 0.017). In vitro approaches were used to investigate the role of miR-195 in EMT and metastasis of PCa. Overexpressed miR-195 in PCa cells inhibited EMT and cell metastasis. Luciferase reporter assays and Western blot analysis were conducted to identify miR-195 targets; results showed that fibroblast growth factor 2 (FGF2) is a direct target. FGF2 was then knocked down in PCa cells and effects similar to those of miR-195 overexpression were observed. Furthermore, the restored FGF2 levels in cells which overexpressed miR-195 likely inhibited the effects of miR-195. And data from ONCOMINE prostate cancer database showed PCa patients with high FGF2 expression showed shorter RFS time (P = 0.046). These findings suggested that miR-195 plays an important role in PCa metastasis.

## Materials and Methods

### Data mining and bioinformatics analysis

Microarray datasets for prostate cancer were retrieved from ONCOMINE Cancer Profiling Database (https://www.oncomine.org/resource/main.html#a%3A1878%3Bd%3A34%3Bdso%3AdatasetName%3Bdt%3ApredefinedClass%3Bec%3A%5B2%5D%3Bepv%3A150001.151078%2C3508%3Bet%3Anone%3Bp%3A200001310%3Bpg%3A1%3Bpvf%3A59119%3Bscr%3Adatasets%3Bss%3Aanalysis%3Bv%3A18 and https://www.oncomine.org/resource/main.html#a%3A8158%3Bd%3A156636663%3Bdso%3AdatasetName%3Bdt%3ApredefinedClass%3Bec%3A%5B2%2C1%2C3%2C5%5D%3Bepv%3A150001%3Bet%3Anone%3Bp%3A200011396%3Bpg%3A1%3Bpvf%3A800075355%2C800075356%3Bscr%3Adatasets%3Bss%3Aanalysis%3Bv%3A18) to investigate FGF2 expression in prostate cancer. Expression of miR-195 in prostate cancer cell lines were retrieved from database (GSE21032, http://www.ncbi.nlm.nih.gov/geo/query/acc.cgi?acc=GSE21032) [24]. miR-195 expression between localised PCa and metastatic PCa and the corresponding biochemical relapse-free time after radical prostatectomy was re-analysed in the database. ROC curves were generated, and AUC was considered to evaluate the sensitivity and specificity of the use of miR-195 expression to diagnose metastatic PCa. FGF2 expression and the corresponding biochemical relapse-free time after radical prostatectomy in prostate cancer was investigated in Cancer Profiling Database.

### Cell culture

LnCap cell line was obtained from American Type Culture Collection (ATCC), DU-145 and PC-3 cell lines were purchased from Shanghai Cell Bank, Chinese Academy of Sciences. LnCap and DU-145 cells were maintained in Dulbecco’s modified Eagle’s medium (DMEM, HyClone, Beijing, China) supplemented with 10% foetal bovine (HyClone, Gaithersburg, Maryland, USA), 100 U/mL penicillin and 100 μg/mL streptomycin (HyClone, Beijing, China). PC-3 cells were cultured in DMEM/F12 medium (HyClone, Beijing, China) supplemented with 10% foetal bovine (HyClone, Gaithersburg, Maryland, USA), 100 U/mL penicillin and 100 μg/mL streptomycin (HyClone, Beijing, China). Cell cultures were incubated in a humidified atmosphere of 95% air and 5% CO_2_ at 37°C.

### Oligonucleotides and cell transfection

miRNA mimic oligonucleotide duplexes and small interfering RNA (siRNA) were chemically synthesised by GenePharma (Shanghai, China). The sense and anti-sense sequences of the hsa-miR-195 mimics were: 5′-UAGCAGCACAGAAAUAUUGGC-3′ and 5′- CAAUAUUUCUGUGCUGCUAUU-3′, respectively. RNA with no homology to any human genomic sequence was used as negative control (NC): 5′-UUCUCCGAACGUGUCACGUTT-3′ (sense) and 5′-ACGUGACACGUUCGGAGAATT-3′ (anti-sense). The sequence of miR-195 siRNA was 5′-GCCAAUAUUUCUGUGCUGCUA-3′ and the control sequence was 5′- CAGUACUUUUGUGUAGUACAA -3′. The sense and anti-sense sequences of FGF2 siRNA were 5′-GGAGUGUGUGCUAACCGUUtt-3′ and 5′- AACGGUUAGCACACACUCCtt-3′, respectively. In cell transfection, cells were seeded in six-well plates and cultured until 50% to 70% confluency was reached in 1 d. Transfection was performed with Lipofectamine 2000 (Invitrogen, Carlsbad, New Mexico, USA) according to the manufacturer’s instructions. The transfection mixture was replaced with a medium containing 10% foetal bovine serum (FBS) after 6 h to 8 h.

### Cell migration and invasion assays

Cell migration and invasion assays were evaluated using a Transwell chamber (Millipore, Billerica, MA, USA). Cells were harvested at 48 h posttransfection, and 5 × 10^4^ cells with 200 μl of serum-free medium were seeded in the upper chamber. The lower chamber was filled with medium supplemented with 10% FBS. In invasion assays, the upper chambers were pre-coated with Matrigel (BD Transduction Laboratories, Franklin Lakes, NJ, USA) before cell seeding was performed. Cells remaining on the upper membrane were removed after 24 h for invasive cells and 12 h for migratory cells. Migratory and invasive cells on the bottom surface were fixed in 90% alcohol and stained with 0.1% crystal violet. Five random fields from each membrane were counted. Experiments were performed independently in triplicate.

### Plasmid construction and luciferase assay

FGF2 3′-UTR-luciferase reporter vectors were created by ligating the FGF2 3′-UTR PCR products into XhoI and NotI restriction sites of the psiCHECK-2™ Vector (Promega, Madison, Wisconsin, USA). Mutant 3′-UTR regions were chemically synthesised and ligated into psiCHECK-2™ vector. Cells were cultured in 24-well plates and each well was transfected with 250 ng of vectors with 50 nM miR-195 or NC. After 48 h of co-transfection, luciferase activity was determined measured using a dual-luciferase reporter assay system (Promega, Madison, Wisconsin, USA) according to the manufacturer’s instructions.

### Western blot analysis

Cells were lysed by RIPA buffer (Beyotime, Shanghai, China) supplemented with protease inhibitors. The protein samples were electrophoresed in 10% sodium dodecyl sulphate polyacrylamide gels. Electrophoresed proteins were transferred to a polyvinylidene fluoride membrane (Millipore, Billerica, Massachusetts, USA) and blocked for 1 h with 5% skim milk at room temperature. After the proteins were incubated with primary antibodies at 4°C overnight, the blots were washed, incubated with horseradish peroxidase (HRP)-labelled secondary antibody at 37°C for 1 h and visualised through enhanced chemiluminescence. Protein levels were determined by normalising against glyceraldehyde 3-phosphate dehydrogenase (GAPDH). The related antibodies were rabbit anti-GAPDH (1:500, Xianzhi Biotechnology, Hangzhou, China), anti-vimentin (1:1000, Proteintech, Chicago, USA), anti-N-cadherin (1:200, Proteintech, Chicago, USA), anti-E-cadherin (1:500, Proteintech, Chicago, USA), mouse anti-FGF2 (1:500, BD Transduction Laboratories, Franklin Lakes, NJ, USA), HRP-labelled goat anti-rabbit secondary antibody (1:3000, Zhongshan Goldenbridge Biotechnology, Beijing, China) and HRP-labelled goat anti-mouse secondary antibody (1:3000, Zhongshan Goldenbridge Biotechnology, Beijing, China).

## Statistical analysis

miR-195 expression and clinical patient data were downloaded from the MSKCC database (http://www.mskcc.org). FGF2 expression and clinical patient data were downloaded from the ONCOMINE Cancer Profiling Database (www.oncomine.org). All experiments above were repeated three times. All of the data in this study were presented as mean ± standard deviation. Statistical calculations were performed using SPSS 16.0. Differences between groups were calculated by *t*-tests or one-way ANOVA. Log-cox test was chosen to perform survival analysis. P < 0.05 was considered statistically significant.

## Results

### miR-195 expression and its effect on the diagnosis and outcome of patients with PCa

**Fig 1:**
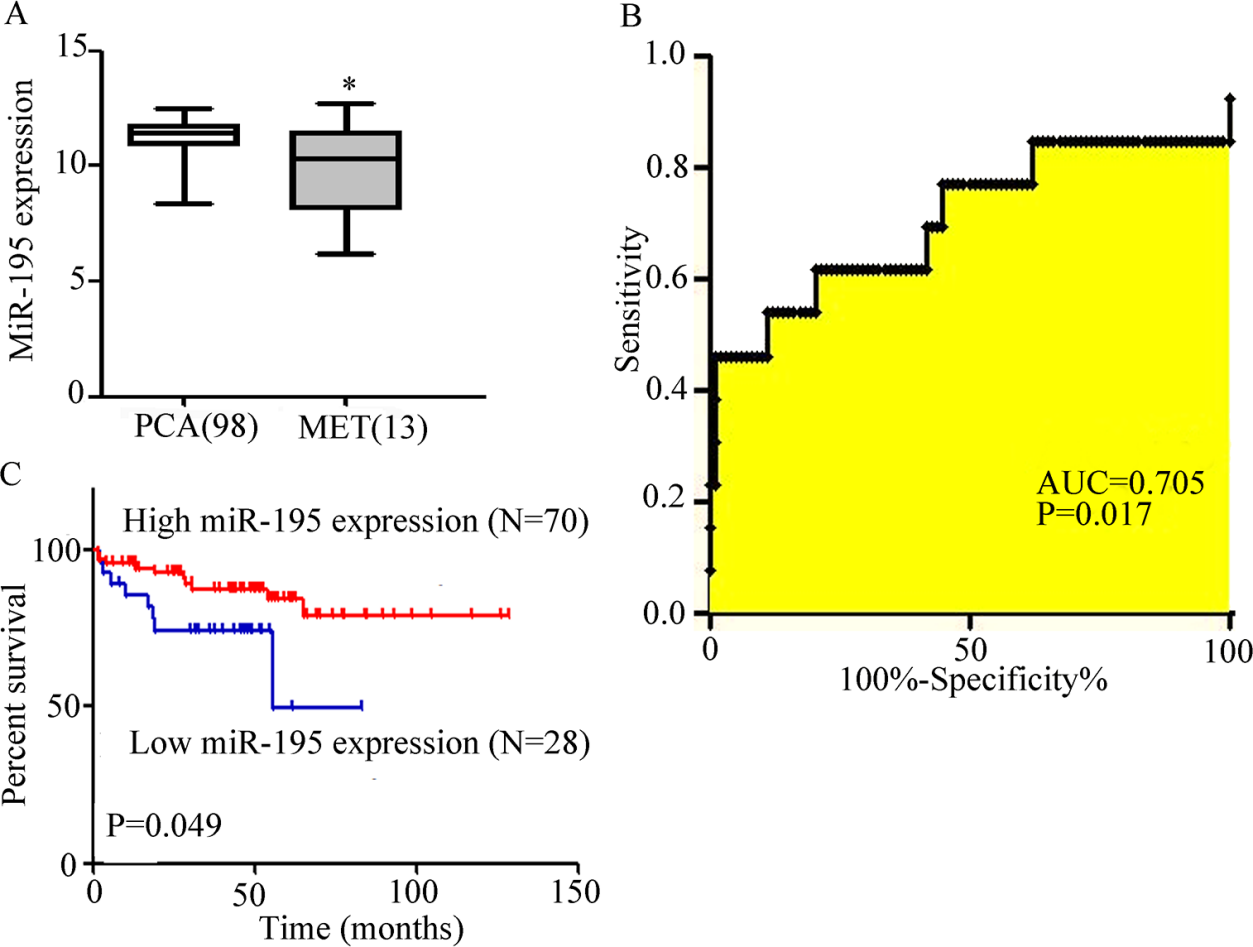
miR-195 expression and impact on diagnosis and outcome of patients with PCa. A) Data in the re-analysed MSKCC prostate cancer database showed that miR-195 is expressed poorly metastatic PCa (metastatic PCa vs. localised PCa: 9.92 ± 0.56 vs. 11.27 ± 0.07, P < 0.0001). B) ROC analysis using expression levels of miR-195 in metastatic PCa and localised PCa (AUC = 0.705, P = 0.017). C) Kaplan–Meier curves of RFS of patients with PCa stratified by the tissues of miR-195 levels. Patients with low miR-195 levels exhibited shorter RFS than those with high levels (P < 0.05) *P < 0.05.

We re-analysed the RNA sequencing data of PCa database (GSE21032). The expression of miR-195 between localised and metastatic PCas was calculated by t-tests. The result showed that miR-195 in metastatic PCa was significantly lower than that in localised PCa (9.92 ± 0.56 vs. 11.27 ± 0.07, P < 0.0001, Fig 1A). Moreover, ROC analysis demonstrated that miR-195 could discriminate between metastatic cancer and primary PCa (AUC = 0.705, P = 0.017, Fig 1B). Therefore, we investigated the effect of low miR-195 expression on RFS in localised PCa. Patients were divided into low and high expression groups by using the number located in 30% of all number, 11.05 as a cut-off of miR-195 expression. RFS analysis was performed in 98 localised PCa cases with follow-up data. Kaplan–Meier curves of RFS showed that PCa patients with low miR-195 expression showed shorter RFS time (P = 0.047, Fig 1C). Hence, miR-195 may play important functions in PCa metastasis.

### EMT inhibition by miR-195 in PCa cells

**Fig 2:**
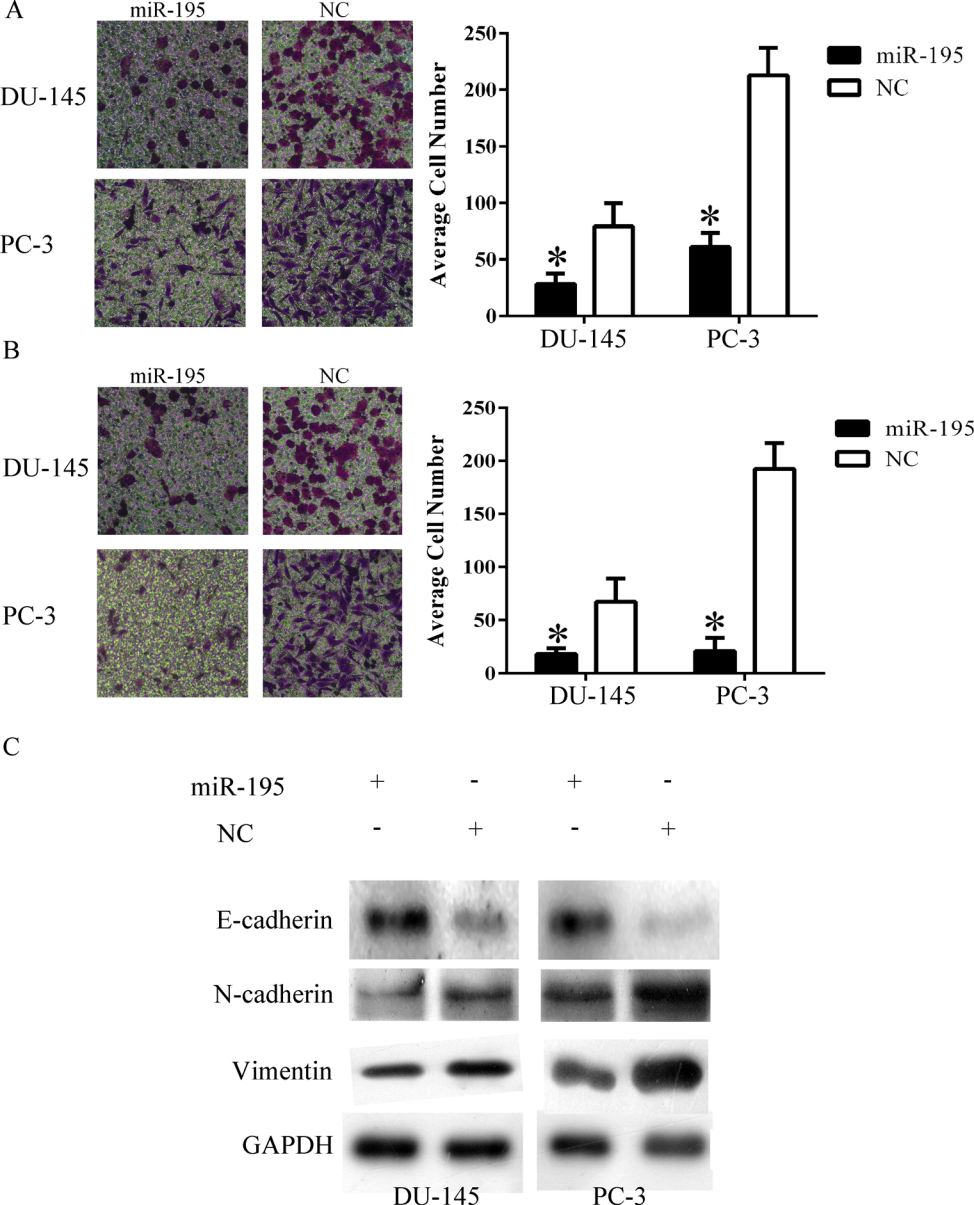
miR-195 inhibited EMT in PCa cells. A) Forced expression of miR-195 inhibited migration in PCa cell lines. B) Forced expression of miR-195 inhibited invasion in PCa cell lines. C) Common EMT markers expression after transfecting miR-195. *P<0.05. Original magnification ×200.

To investigate whether miR-195 affects PCa cell invasion and migration, we transfected miR-195 inhibitor or control inhibitor in LnCap cells and miR-195 mimics or control miRNA in DU-145 and PC-3 cells and analysed cell migration and invasion ability by using Transwell. The results showed that the miR-195 mimics group contained fewer numbers of cells, which migrated into the lower chamber of the Transwell filter, than the miR-NC group in Du-145 and PC-3 cells (Fig 2A). Invasion ability was also significantly downregulated in miR-195-transfected DU-145 and PC-3 cells (Fig 2B). However, no significant different was observed between LnCap cells transfected miR-195 inhibitor and control inhibitor in invasion and migration assays (S1 Fig). So we chose Du-145 and PC-3 for the next experiments. EMT can change cell invasion and migration ability. To identify whether miR-195 can affect EMT, we evaluated some common EMT markers through Western blot analysis. A decreased expression of vimentin and N-cadherin proteins was observed in cells transfected with miR-195 mimics compared with that in cells transfected with control miRNA. E-cadherin expression increased in cells transfected with miR-195 mimics (Fig 2C). These results suggested that miR-195 may impede cell invasion and migration by inhibiting EMT.

### FGF2 was a direct target of miR-195

**Fig 3:**
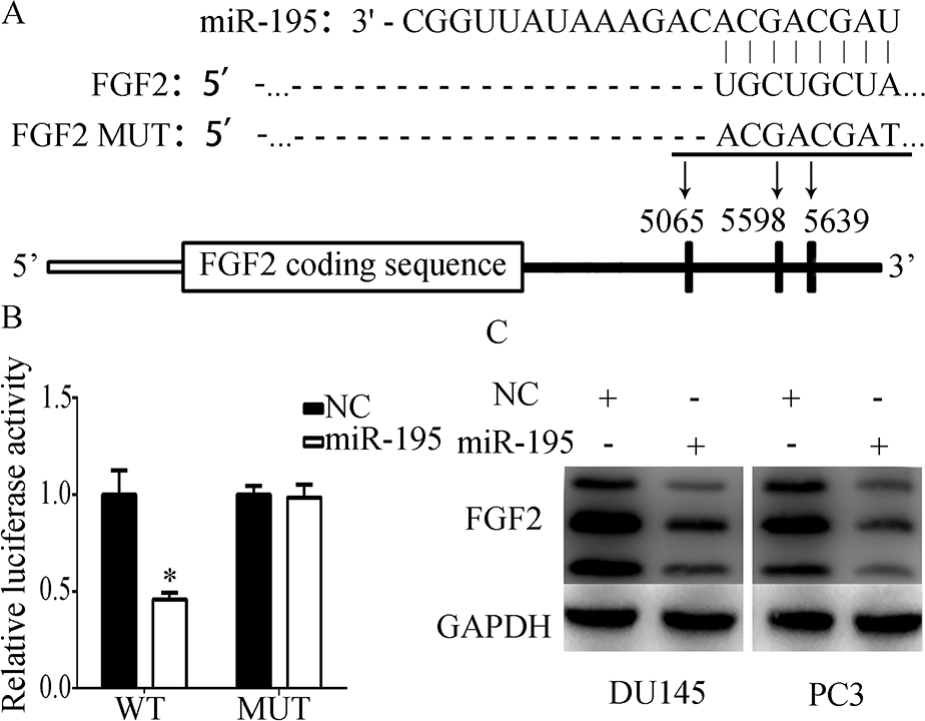
FGF2 was a direct target of miR-195 A) Sequence alignment of human miR-195 with 3’ UTR of FGF2 predicted by DIANA LAB. B) Dual-luciferase assay results for PC-3 cells showed that miR-195 upregulation decreased luciferase activity. C) FGF2 protein expression in PCa cell lines was determined through Western blot analysis at 72 h post-transfection. GAPDH was used as a control. *P<0.05.

Bioinformatics analyses were performed using DIANA microT v5.0 and TargetScanHuman 6.2 to identify the target genes of miR-195. FGF2 was chosen as the potential target gene because it was predicted in both databases and had the highest Total context score in TargetScanHuman 6.2. 3′-UTRs of FGF2 were predicted to contain five binding sites for miR-195. We analysed the sequence of binding sites and found that the sites located from 1053 to 1059 nucleotides and 1113 to 1119 nucleotides were a part of other three binding sites (Table 1). Therefore, only the sites of 5065 to 5072, 5598 to 5605, 5639 to 5646, as well as mutation sites, were cloned into luciferase reporter plasmids (Fig 3A). The reporter plasmids were co-transfected with miR-195 mimics or NC in PC-3 cells. Luciferase activity assay showed that miR-195 mimics significantly suppressed the luciferase activity of the reporter plasmid containing binding sites compared with the NC of the wild-type reporter but not of the mutant one (Fig 3B). miR-195 overexpression remarkably inhibited FGF2 expression in DU-145 and PC-3 cells (Fig 3C). These results suggested that FGF2 is a direct target gene of miR-195.

**Table 1:** Predicted binding sites of miR-195 for FGF2.

**Transcript position**	**Seed sequence**	**Conservation**
1053–1059	CGACGAU	4
1113–1119	ACGACGA	2
5065–5072	ACGACGAU	3
5598–5605	ACGACGAU	8
5639–5646	ACGACGAU	9

### FGF2 knockdown elicited similar effects on PCa cells with overexpressed miR-195

**Fig 4:**
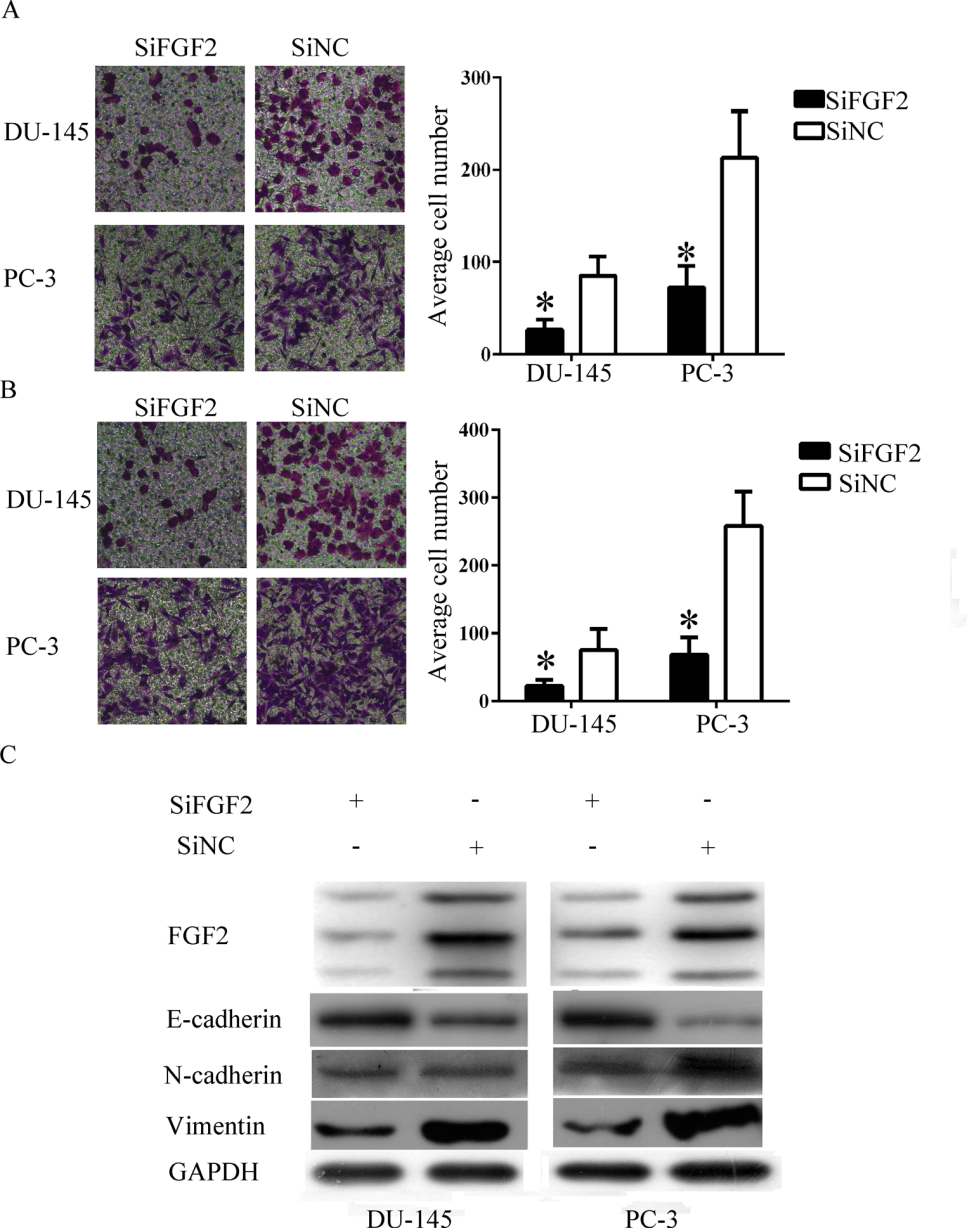
FGF2 knockdown resulted similar effects in PCa cells with overexpress miR-195 A and B) Low expression of FGF2 inhibited migration and invasion in PCa cell lines. C) FGF2 and common EMT markers expression after siFGF2 was transfected. *P < 0.05.

FGF2 was knocked down in PCa cells by introducing siRNA to determine whether the effects of miR-195 can be partly explained by targeting of FGF2. The FGF2 expression reduced at 72 h posttransfection, as detected by Western blot analysis (Fig 4C). The number of migrated cells was significantly lower in DU-145 and PC-3 cells transfected with FGF2 siRNA than transfected with control siRNA (Fig 4A). The number of invaded cells was also reduced (Fig 4B). Consistent with miR-195 transfection, the inhibition of FGF2 expression in PCa cells decreased vimentin and N-cadherin expression and increased E-cadherin expression (Fig 4C).

### miR-195 inhibited EMT in an FGF2 dependent manner

**Fig 5:**
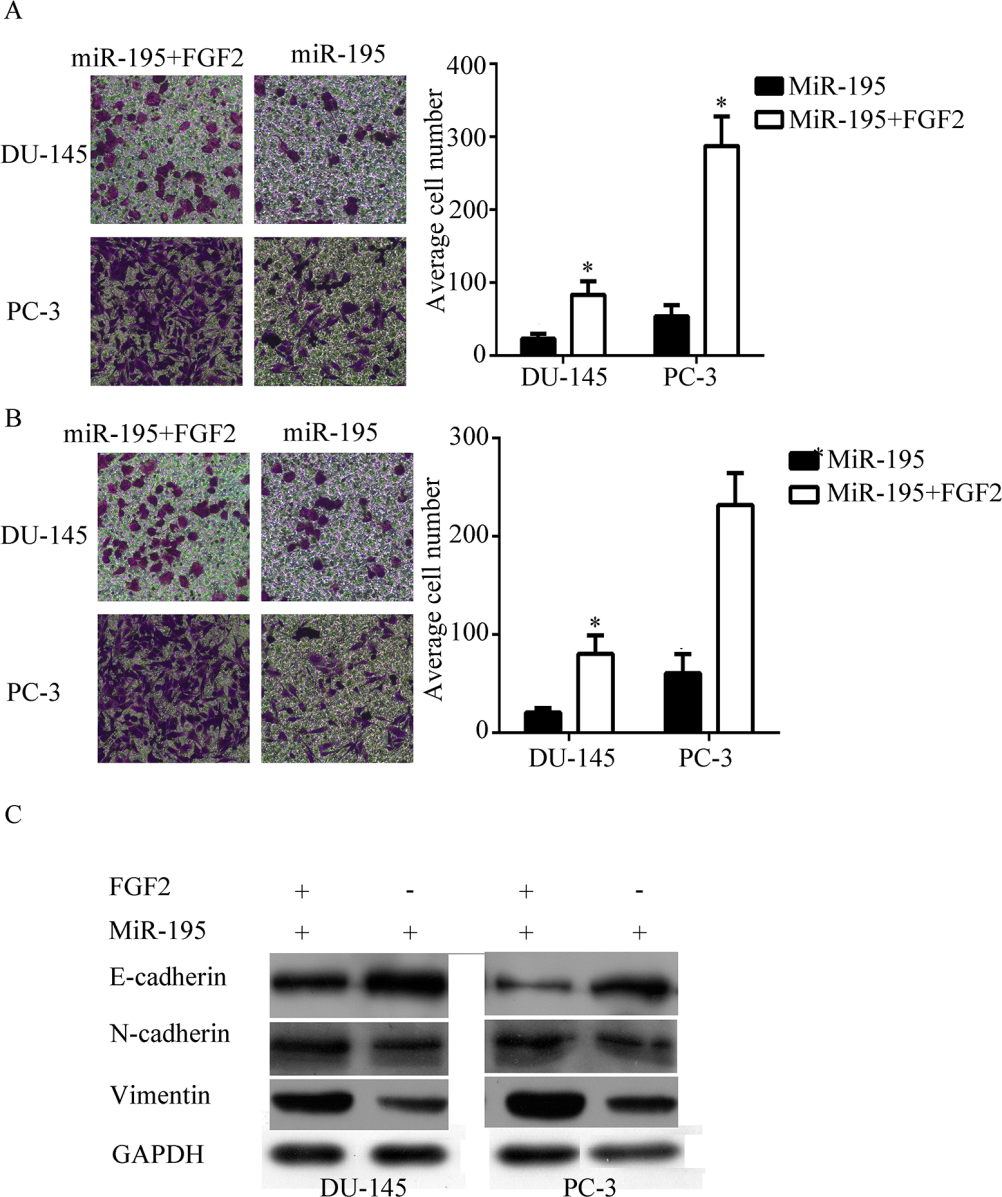
miR-195 inhibited EMT in an FGF2-dependent manner. PCa cell lines were treated with recombinant human FGF2 protein after transfection with miR-195 was performed. A) The number of migrated cells increased after treatment. B) The number of invaded cells increased after treatment. C) Common EMT markers expression after 48 h of treatment.

To further confirm whether FGF2 was required for miR-195 mediated EMT, we treated the cells with recombinant human FGF2 protein (Sino Biological Inc., Beijing, China) after transfection was performed. The number of migrated and invaded cells significantly increased after treatment was administered (Fig 5A and 5B). Vimentin and N-cadherin expression increased, whereas E-cadherin expression decreased after 48 h of treatment (Fig 5C). These results showed that miR-195 inhibited EMT of PCa cells in an FGF2-dependent manner.

### FGF2 expression and its effect on the outcome of patients with PCa

**Fig 6:**
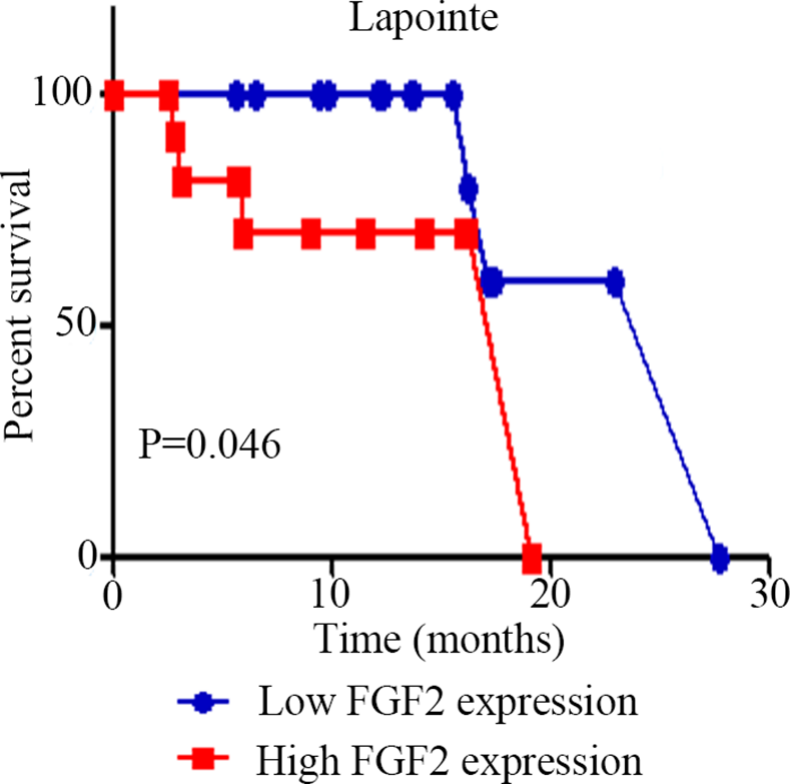
FGF2 expression impact outcome of patients with PCa. Kaplan–Meier curves of RFS of patients with PCa stratified by the tissues of FGF2 levels. In Lapointe’s study patients with high FGF2 levels exhibited shorter RFS than those with high levels (P < 0.05).

ONCOMINE prostate cancer database is the most famous database in the world with several high quality datasets. In Lapointe prostate cancer dataset, FGF2 expression had no statistically significant between metastatic cancer and primary PCa tissues (-0.91190 ± 0.62090 vs. 0.57764 ± 0.35019, P = 0 .174). Primary PCa patients were divided into low and high expression groups by using the median number as a cut-off of FGF2 expression. Kaplan–Meier curves of RFS indicated that PCa patients with high FGF2 expression showed shorter RFS time (P = 0.046, Fig 6).

## Discussion

miRNAs are abnormally expressed in PCa and changes in miRNA expression affect the onset, development and metastasis of PCa. For example, miR-146a suppresses tumour growth and progression in CRPC [25]. miR-29b, miR-200 family and miR-205 influence PCa metastasis by regulating EMT [11, 13]. Furthermore, miRNA expression can be used for diagnosis, staging and prognosis analysis [26]. In this study, re-analysis of the data obtained from MSKCC showed that miR-195 was poorly expressed in metastatic PCa. ROC analysis demonstrated that miR-195 can discriminate metastatic cancer from primary PCa; low miR-195 expression exhibited shorter RFS time. These results showed that miR-195 may be implicated in PCa metastasis; miR-195 can be used as a biomarker for diagnosis and prognosis analysis.

Metastatic disease is responsible for the majority of cancer-related deaths. The conversion of primary tumour to metastatic cancer is a multistep process. EMT is an essential process during metastasis. Therefore, it is reasonable to expect that miRNAs may control metastasis by regulating EMT. A great numbers of miRNAs are found play integral roles in modulating EMT [10], miR-195 is also correlated with lymph node metastasis and poor prognosis in colorectal cancer [27]. However, the regulatory procedure of miR-195 in PCa remains unclear. In our study, the effects of miR-195 on the regulation of EMT and metastasis of PCa were investigated. The induction of miR-195 expression inhibited EMT and invasiveness of PCa cells.

miRNAs perform biological functions by directly binding to the 3′-UTR of mRNAs and by impairing protein translation. As such, genes were regulated by miR-195. CARMA3 and Bcl-2 are direct targets of miR-195 in colorectal cancer [18, 28]. IGF1R and HDGF are direct targets of miR-195 in NSCLC [14, 17].In this study, FGF2 was confirmed as a direct target gene of miR-195 through luciferase assay and Western blot analysis. Besides that, high FGF2 expression exhibited shorter RFS time in PCa patients. FGF2, which belongs to the 23-member FGF family, was first found in 1974 [29]. FGF2 is regarded as a prototypic growth factor [30]. FGF2 is also overexpressed in a great numbers of human carcinomas, including PCa, and implicated in oncogenic behaviours, including invasion and migration [31–34]. FGF2 expression is regulated by many miRNAs. FGF2 is also a target of miR-503 in hepatocellular carcinomas [35]. miR-646 can downregulate FGF2 and suppress osteosarcoma cell metastasis [36]. In NSCLC, FGF2 is a target of miR-152 [37]. FGF2 also induces EMT in many types of cells, such as Hertwig’s epithelial root sheath (HERS) cells, renal tubular cells, colon cancer cells and PCa cells [38–41]. FGF2 induces EMT through a serious of signalling pathways [38, 41, 42]. In HERS cells, TGF-β1 and FGF2 induce EMT through a MAPK/ERK-dependent signalling pathway [38]. In PC-3 cells, AKT/GSK-3β signalling pathway affects EMT, which is promoted by FGF2, by controlling stability, localisation and transcription of Snail [41]. FGF2 also promotes EMT through PI 3-kinase signalling pathway [42]. In this study, our data showed that the knockdown of FGF2 elicited similar effects on PCa cells with overexpressed miR-195 by inhibiting EMT and invasiveness. After miR-195 was transfected, the treated cells with recombinant human FGF2 protein abrogated the effects of miR-195.

In conclusion, miR-195 inhibited PCa cell metastasis and EMT by targeting FGF2. miR-195 restoration may provide new therapeutic methods to treat metastatic PCa.

## Supporting Information

S1 Fig The effect of miR-195 inhibitor in LnCap. Transfected miR-195 inhibitor in LnCap cells didn’t affect the migration and invasion abilities.
(TIF)

S1 File Clinical Studies Checklist. (DOCX)

## References

[1] Siegel R, Ma J, Zou Z, Jemal A. Cancer statistics, 2014. CA: a cancer journal for clinicians. 2014;64(1):9–29. 10.3322/caac.21208 . 24399786

[2] Jilg CA, Ketscher A, Metzger E, Hummel B, Willmann D, Russeler V, et al PRK1/PKN1 controls migration and metastasis of androgen-independent prostate cancer cells. Oncotarget. 2014 . 25504435 10.18632/oncotarget.2653 PMC4350344

[3] Gupta GP, Massague J. Cancer metastasis: building a framework. Cell. 2006;127(4):679–95. 10.1016/j.cell.2006.11.001 . 17110329

[4] Semenas J, Allegrucci C, Boorjian SA, Mongan NP, Persson JL. Overcoming drug resistance and treating advanced prostate cancer. Current drug targets. 2012;13(10):1308–23. 22746994 10.2174/138945012802429615 PMC3474961

[5] Valastyan S, Weinberg RA. Tumor metastasis: molecular insights and evolving paradigms. Cell. 2011;147(2):275–92. 10.1016/j.cell.2011.09.024 22000009 PMC3261217

[6] De Craene B, Berx G. Regulatory networks defining EMT during cancer initiation and progression. Nature reviews Cancer. 2013;13(2):97–110. 10.1038/nrc3447 . 23344542

[7] Thiery JP, Acloque H, Huang RY, Nieto MA. Epithelial-mesenchymal transitions in development and disease. Cell. 2009;139(5):871–90. 10.1016/j.cell.2009.11.007 19945376

[8] Lamouille S, Xu J, Derynck R. Molecular mechanisms of epithelial-mesenchymal transition. Nature reviews Molecular cell biology. 2014;15(3):178–96. 10.1038/nrm3758 24556840 PMC4240281

[9] Vidigal JA, Ventura A. The biological functions of miRNAs: lessons from in vivo studies. Trends in cell biology. 2014 10.1016/j.tcb.2014.11.004 . 25484347 PMC4344861

[10] Diaz-Lopez A, Moreno-Bueno G, Cano A. Role of microRNA in epithelial to mesenchymal transition and metastasis and clinical perspectives. Cancer management and research. 2014;6:205–16. 10.2147/CMAR.S38156 24812525 PMC4008290

[11] Ru P, Steele R, Newhall P, Phillips NJ, Toth K, Ray RB. miRNA-29b suppresses prostate cancer metastasis by regulating epithelial-mesenchymal transition signaling. Molecular cancer therapeutics. 2012;11(5):1166–73. 10.1158/1535-7163.MCT-12-0100 . 22402125

[12] Zhang J, Zhang H, Liu J, Tu X, Zang Y, Zhu J, et al miR-30 inhibits TGF-beta1-induced epithelial-to-mesenchymal transition in hepatocyte by targeting Snail1. Biochem Biophys Res Commun. 2012;417(3):1100–5. 10.1016/j.bbrc.2011.12.121 . 22227196

[13] Gregory PA, Bert AG, Paterson EL, Barry SC, Tsykin A, Farshid G, et al The miR-200 family and miR-205 regulate epithelial to mesenchymal transition by targeting ZEB1 and SIP1. Nature cell biology. 2008;10(5):593–601. 10.1038/ncb1722 . 18376396

[14] Wang X, Wang Y, Lan H, Li J. MiR-195 inhibits the growth and metastasis of NSCLC cells by targeting IGF1R. Tumour biology: the journal of the International Society for Oncodevelopmental Biology and Medicine. 2014;35(9):8765–70. 10.1007/s13277-014-2140-5 . 24874051

[15] Lin Y, Wu J, Chen H, Mao Y, Liu Y, Mao Q, et al Cyclin-dependent kinase 4 is a novel target in micoRNA-195-mediated cell cycle arrest in bladder cancer cells. FEBS letters. 2012;586(4):442–7. 10.1016/j.febslet.2012.01.027 . 22289176

[16] Deng H, Guo Y, Song H, Xiao B, Sun W, Liu Z, et al MicroRNA-195 and microRNA-378 mediate tumor growth suppression by epigenetical regulation in gastric cancer. Gene. 2013;518(2):351–9. 10.1016/j.gene.2012.12.103 . 23333942

[17] Guo H, Li W, Zheng T, Liu Z. MiR-195 targets HDGF to inhibit proliferation and invasion of NSCLC cells. Tumour biology: the journal of the International Society for Oncodevelopmental Biology and Medicine. 2014;35(9):8861–6. 10.1007/s13277-014-2153-0 . 24891187

[18] Wang L, Qian L, Li X, Yan J. MicroRNA-195 inhibits colorectal cancer cell proliferation, colony-formation and invasion through targeting CARMA3. Molecular medicine reports. 2014;10(1):473–8. 10.3892/mmr.2014.2178 . 24787958

[19] Yang Y, Li M, Chang S, Wang L, Song T, Gao L, et al MicroRNA-195 acts as a tumor suppressor by directly targeting Wnt3a in HepG2 hepatocellular carcinoma cells. Molecular medicine reports. 2014;10(5):2643–8. 10.3892/mmr.2014.2526 . 25174704

[20] Zhao FL, Dou YC, Wang XF, Han DC, Lv ZG, Ge SL, et al Serum microRNA-195 is down-regulated in breast cancer: a potential marker for the diagnosis of breast cancer. Molecular biology reports. 2014;41(9):5913–22. 10.1007/s11033-014-3466-1 . 25103018

[21] Amer M, Elhefnawi M, El-Ahwany E, Awad AF, Gawad NA, Zada S, et al Hsa-miR-195 targets PCMT1 in hepatocellular carcinoma that increases tumor life span. Tumour biology: the journal of the International Society for Oncodevelopmental Biology and Medicine. 2014;35(11):11301–9. 10.1007/s13277-014-2445-4 . 25119594

[22] Mao JH, Zhou RP, Peng AF, Liu ZL, Huang SH, Long XH, et al microRNA-195 suppresses osteosarcoma cell invasion and migration in vitro by targeting FASN. Oncology letters. 2012;4(5):1125–9. 10.3892/ol.2012.863 23162665 PMC3499598

[23] Porkka KP, Pfeiffer MJ, Waltering KK, Vessella RL, Tammela TL, Visakorpi T. MicroRNA expression profiling in prostate cancer. Cancer research. 2007;67(13):6130–5. 10.1158/0008-5472.CAN-07-0533 . 17616669

[24] Taylor BS, Schultz N, Hieronymus H, Gopalan A, Xiao Y, Carver BS, et al Integrative genomic profiling of human prostate cancer. Cancer cell. 2010;18(1):11–22. 10.1016/j.ccr.2010.05.026 20579941 PMC3198787

[25] Xu B, Wang N, Wang X, Tong N, Shao N, Tao J, et al MiR-146a suppresses tumor growth and progression by targeting EGFR pathway and in a p-ERK-dependent manner in castration-resistant prostate cancer. The Prostate. 2012;72(11):1171–8. 10.1002/pros.22466 . 22161865

[26] Wen X, Deng FM, Wang J. MicroRNAs as predictive biomarkers and therapeutic targets in prostate cancer. American journal of clinical and experimental urology. 2014;2(3):219–30. 25374924 PMC4219315

[27] Wang X, Wang J, Ma H, Zhang J, Zhou X. Downregulation of miR-195 correlates with lymph node metastasis and poor prognosis in colorectal cancer. Medical oncology. 2012;29(2):919–27. 10.1007/s12032-011-9880-5 . 21390519

[28] Liu L, Chen L, Xu Y, Li R, Du X. microRNA-195 promotes apoptosis and suppresses tumorigenicity of human colorectal cancer cells. Biochem Biophys Res Commun. 2010;400(2):236–40. 10.1016/j.bbrc.2010.08.046 . 20727858

[29] Gospodarowicz D, Jones KL, Sato G. Purification of a growth factor for ovarian cells from bovine pituitary glands. Proceedings of the National Academy of Sciences of the United States of America. 1974;71(6):2295–9. 4526208 10.1073/pnas.71.6.2295 PMC388439

[30] Cronauer MV, Schulz WA, Seifert HH, Ackermann R, Burchardt M. Fibroblast growth factors and their receptors in urological cancers: basic research and clinical implications. European urology. 2003;43(3):309–19. . 12600436 10.1016/s0302-2838(03)00005-8

[31] Turner N, Grose R. Fibroblast growth factor signalling: from development to cancer. Nature reviews Cancer. 2010;10(2):116–29. 10.1038/nrc2780 . 20094046

[32] Giri D, Ropiquet F, Ittmann M. Alterations in expression of basic fibroblast growth factor (FGF) 2 and its receptor FGFR-1 in human prostate cancer. Clinical cancer research: an official journal of the American Association for Cancer Research. 1999;5(5):1063–71. . 10353739

[33] Farhat FS, Tfayli A, Fakhruddin N, Mahfouz R, Otrock ZK, Alameddine RS, et al Expression, prognostic and predictive impact of VEGF and bFGF in non-small cell lung cancer. Critical reviews in oncology/hematology. 2012;84(2):149–60. 10.1016/j.critrevonc.2012.02.012 . 22494932

[34] Nomura S, Yoshitomi H, Takano S, Shida T, Kobayashi S, Ohtsuka M, et al FGF10/FGFR2 signal induces cell migration and invasion in pancreatic cancer. British journal of cancer. 2008;99(2):305–13. 10.1038/sj.bjc.6604473 18594526 PMC2480967

[35] Zhou B, Ma R, Si W, Li S, Xu Y, Tu X, et al MicroRNA-503 targets FGF2 and VEGFA and inhibits tumor angiogenesis and growth. Cancer letters. 2013;333(2):159–69. 10.1016/j.canlet.2013.01.028 . 23352645

[36] Sun XH, Geng XL, Zhang J, Zhang C. miRNA-646 suppresses osteosarcoma cell metastasis by downregulating fibroblast growth factor 2 (FGF2). Tumour biology: the journal of the International Society for Oncodevelopmental Biology and Medicine. 2014 10.1007/s13277-014-2822-z . 25403884

[37] Cheng Z, Ma R, Tan W, Zhang L. MiR-152 suppresses the proliferation and invasion of NSCLC cells by inhibiting FGF2. Experimental & molecular medicine. 2014;46:e112 10.1038/emm.2014.51 25190353 PMC4150934

[38] Chen J, Chen G, Yan Z, Guo Y, Yu M, Feng L, et al TGF-beta1 and FGF2 stimulate the epithelial-mesenchymal transition of HERS cells through a MEK-dependent mechanism. Journal of cellular physiology. 2014;229(11):1647–59. 10.1002/jcp.24610 . 24610459

[39] Masola V, Gambaro G, Tibaldi E, Brunati AM, Gastaldello A, D'Angelo A, et al Heparanase and syndecan-1 interplay orchestrates fibroblast growth factor-2-induced epithelial-mesenchymal transition in renal tubular cells. The Journal of biological chemistry. 2012;287(2):1478–88. 10.1074/jbc.M111.279836 22102278 PMC3256891

[40] Sakuma K, Aoki M, Kannagi R. Transcription factors c-Myc and CDX2 mediate E-selectin ligand expression in colon cancer cells undergoing EGF/bFGF-induced epithelial-mesenchymal transition. Proceedings of the National Academy of Sciences of the United States of America. 2012;109(20):7776–81. 10.1073/pnas.1111135109 22547830 PMC3356678

[41] Liu ZC, Wang HS, Zhang G, Liu H, Chen XH, Zhang F, et al AKT/GSK-3beta regulates stability and transcription of snail which is crucial for bFGF-induced epithelial-mesenchymal transition of prostate cancer cells. Biochimica et biophysica acta. 2014;1840(10):3096–105. 10.1016/j.bbagen.2014.07.018 . 25088797

[42] Lee JG, Kay EP. Cross-talk among Rho GTPases acting downstream of PI 3-kinase induces mesenchymal transformation of corneal endothelial cells mediated by FGF-2. Investigative ophthalmology & visual science. 2006;47(6):2358–68. 10.1167/iovs.05-1490 . 16723445

