# Supplementary figures and images for: miR-195 Inhibits EMT by Targeting FGF2 in Prostate Cancer Cells
Source: PLoS One. 2015 Dec 9;10(12):e0144073. doi: 10.1371/journal.pone.0144073 (PMC4674136; doi:10.1371/journal.pone.0144073)

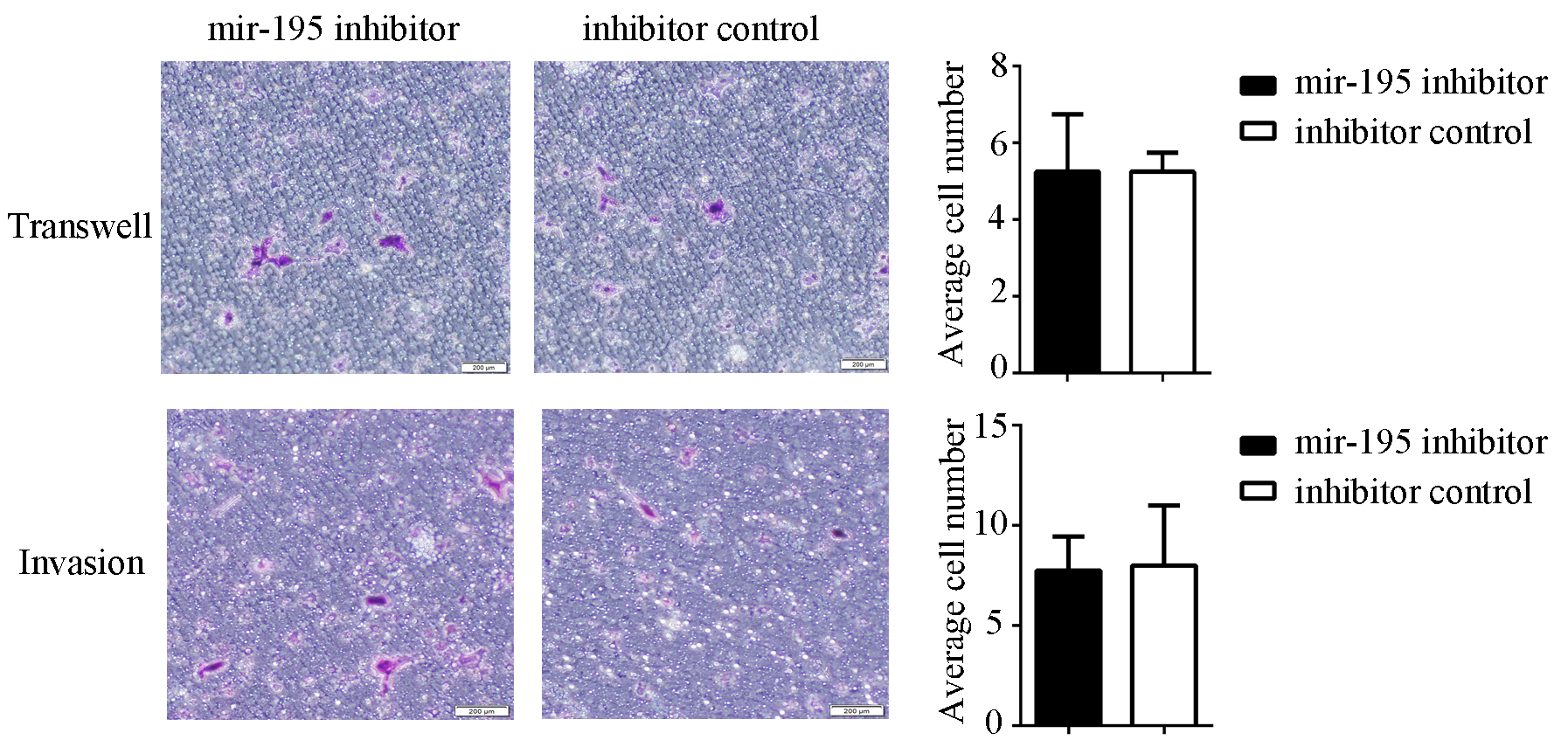

Supplement: S1 Fig — Transfected miR-195 inhibitor in LnCap cells didn’t affect the migration and invasion abilities. (TIF) [file pone.0144073.s001.tif]
