# Supplementary material for: miR-195 Inhibits EMT by Targeting FGF2 in Prostate Cancer Cells
Source: PLoS One. 2015 Dec 9;10(12):e0144073. doi: 10.1371/journal.pone.0144073 (PMC4674136; doi:10.1371/journal.pone.0144073)
Supplement: S1 File — (DOCX) [file pone.0144073.s002.docx]

***PLOS ONE* Clinical Studies Checklist**

1. For at least one author, provide an institutional email address for correspondence.

| Ming Chen : mingchen0712@seu.edu.cn |
| --- |

1. Describe any previous interactions you have had with *PLOS ONE* in relation to this work.

| None |
| --- |

1. If submitting a systematic review or meta-analysis, please describe recent related systematic reviews and meta-analyses, and how the current study adds to the field in the context of previous reports.

| No |
| --- |

1. Please explain the rationale for your study.

| This study is a research article. First, we re-analyzed data from Memorial Sloan Kettering Cancer Center, and found miR-195 was low expressed in metastatic PCa. Then we proved miR-195 inhibited EMT through FGF2 by in vitro experiment |
| --- |

| **HUMAN SUBJECTS RESEARCH**  **Please complete #5-9 if your study involved any human participants or human subjects’ data, including medical record data. These questions should be addressed for prospective and retrospective studies.** | | |
| --- | --- | --- |
| 5. | If you did not have ethics approval, please explain why this was not necessary.   \| **In this study, we just re-analyzed data from MSKCC and ONCOMINE prostate cancer database, we didn’t involve any other human participants or human subjects’ data.** \| \| --- \| | |
| 6. | Upload the letter of ethical approval from your ethics committee as file type “Other”. If the letter is not in English, please include a translation.  ___ Uploaded ___ N/A | |
|  |  | |
| 7. | Upload a copy of the study protocol approved by your ethics committee as file type “Other” – if the protocol is not in English, please include a translation.  ___ Uploaded ___ N/A (if N/A, provide explanation below) | |
|  | \|  \| \| --- \| | |
| 8. | If the study involved patients and/or patient medical data, report the date range within which patients were recruited to the study in the Methods section.  ___ Completed ___ N/A | |
|  |  | |
| 9. | If the study included patients/participants, include details on sample size calculation and power analysis below and in the Methods section.  ___ Completed ___ N/A | |
|  |  | |
| **REPORTING GUIDELINES**  **For the relevant study type, upload the applicable reporting checklist* and other documents listed as Supporting Information files for your submission. Note: in most cases, only one study type will be relevant.** | |  |
| 10. | ***Clinical Trial***   - CONSORT flow diagram - Completed CONSORT or TREND checklist - Include details on clinical trial registration in your Methods section. - Completed TIDieR checklist   ___ Uploaded ___ N/A | |
|  |  | |
| 11. | ***Observational study***   - Completed STROBE checklist   ___ Uploaded ___ N/A | |
|  |  | |
| 12. | - ***Meta-analysis or Systematic Review*** PRISMA flowchart (this should be Figure 1 of your manuscript) - Completed PRISMA checklist   ___ Uploaded ___ N/A | |
|  |  | |

***URLs for reporting checklists and guidelines:**

CONSORT (randomized trials): <http://www.consort-statement.org/consort-statement/checklist>

CONSORT flow diagram: <http://www.consort-statement.org/consort-statement/flow-diagram>

TREND (non-randomized trials): <http://www.cdc.gov/trendstatement/>

STROBE: <http://www.strobe-statement.org/index.php?id=available-checklists>

PRISMA: <http://www.prisma-statement.org/statement.htm>

TIDieR: <http://www.equator-network.org/reporting-guidelines/tidier/>
